# Characterizing candidate decompression rates for hypobaric hypoxic stunning of pigs. Part 2: Pathological consequences

**DOI:** 10.3389/fvets.2022.1027883

**Published:** 2022-11-09

**Authors:** Emma M. Baxter, Dorothy E. F. McKeegan, Marianne Farish, Jill R. Thomson, Richard E. Clutton, Stephen N. Greenhalgh, Rachael Gregson, Jessica E. Martin

**Affiliations:** ^1^Animal and Veterinary Sciences Research Group, Scotland's Rural College (SRUC), Edinburgh, United Kingdom; ^2^School of Biodiversity, One Health and Veterinary Medicine, College of Medical, Veterinary and Life Sciences, University of Glasgow, Glasgow, United Kingdom; ^3^The Wellcome Trust Critical Care Laboratory for Large Animals LARIF, The Roslin Institute, The University of Edinburgh, Edinburgh, United Kingdom; ^4^The Royal (Dick) School of Veterinary Studies and The Roslin Institute, The University of Edinburgh, Edinburgh, United Kingdom; ^5^School of Natural and Environmental Sciences, Newcastle University, Newcastle upon Tyne, United Kingdom

**Keywords:** low atmospheric pressure, slaughter, killing, swine, anatomy, decompression

## Abstract

Pigs are commonly stunned pre-slaughter by exposure to carbon dioxide (CO_2_), but this approach is associated with significant welfare concerns. Hypobaric hypoxia, achieved with gradual decompression (also known as Low Atmospheric Pressure Stunning or LAPS) may be an alternative, allowing the retention of welfare friendly handling approaches and group stunning. Although validated in poultry, the feasibility and welfare consequences of gradual decompression for pigs are unknown. Here, we characterize pathological changes in 60 pigs resulting from exposure to a range of candidate decompression curves (ranging from 40 to 100 ms^−1^ ascent equivalent, with two cycle durations 480 and 720 s). To protect welfare, we worked on unconscious, terminally anesthetized pigs which were subject to detailed post-mortem examinations by a specialized porcine veterinary pathologist. All pigs were killed as a result of exposure to decompression, irrespective of cycle rate or length. Pigs showed no external injuries during ante-mortem inspections. Exposing pigs to decompression and the unavoidable subsequent recompression resulted in generalized congestion of the carcass, organs and body cavities including the ears, oral cavity, conjunctivae and sclera, mucosa of other external orifices (anus and vulva), nasal planum, nasal cavities including nasal conchae, frontal sinuses, cranium, meninges, brain, larynx, trachea, lungs, heart, parietal pleura of the thoracic cavity, peritoneum of the abdominal cavity, stomach, small intestine, caecum, colon, liver, spleen and kidneys and representative joint cavities in the limbs (stifles and elbows). Various severities of hemorrhage were observed in the conjunctivae and sclera, mucosa of other external orifices (anus and vulva), nasal cavities including nasal conchae, frontal sinuses, cranium, meninges, brain, larynx, tracheal lumen, lungs, parietal pleura of the thoracic cavity, liver, spleen and kidneys and representative joint cavities in the limbs (stifles and elbows). In general, faster decompression rates produced higher scores, but in the conjunctivae, sclera and kidneys, faster decompression rates were associated with marginally lower congestion scores. There was considerable individual variation in pathological scores across all body regions. The congestion and hemorrhage observed could translate into welfare harms in conscious pigs undergoing this type of stunning, depending when in the cycle the damage is occurring, but no welfare related conclusions can be drawn from the responses of unconscious pigs. Since recompression is always required, its effects cannot be separated from decompression, however cessation of cardiac activity several minutes before recompression should have eliminated any haemodynamic effects relating to cardiac function and blood pressure. This study represents the first systematic attempt to identify candidate rate profiles to underpin future explorations of decompression as a stunning method for pigs. These pathological findings also inform discussions about the likely carcass quality implications of this novel stunning method.

## Introduction

Carbon dioxide (CO_2_) is commonly employed in commercial pig slaughter as it is reliable, allows pigs to be stunned in groups and enables high-throughput at slaughter plants (1, 2). Controlled atmosphere stunning (CAS) systems in general employ the addition of gases (i.e., CO_2_) and are classed as non-physical methods, which require minimal physical restraint alongside minimal animal and operator interaction, providing an important welfare refinement to electrical stunning methods for both pigs and poultry (2, 3). CAS systems expose animals to gas mixtures which result in induction to loss of consciousness, followed by a hold phase to ensure a non-recovery state. The use of CO_2_, exposes the animal to an increasing hypercapnic hypoxic environment, which induces unconsciousness via suppression of basal and evoked neural activity through widespread intracellular acidosis (3). Critically, this results in a non-immediate stun and instead a gradual loss of conscious, where the animal may be exposed to a noxious environment. There are important welfare concerns associated with the use of CO_2_, and ample evidence of welfare compromise and aversion in pigs on exposure to CO_2_, including avoidance of the environment (even to obtain food after fasting), respiratory difficulties, escape attempts and vocalizations (2, 4–7). In 2003, the Farm Animal Welfare Council (now Animal Welfare Committee) (8) recommended that stunning of pigs with high concentrations of CO_2_ should be discontinued but it remains widely used due to advantages over electrical stunning with respect to pig handling and since no high-throughput or practical (including economic factors) has been identified (2, 9, 10).

Hypobaric hypoxia, also known as Low Atmospheric Pressure Stunning (LAPS), is emerging as a potentially high welfare alternative approach for stunning livestock. Animals are placed in a sealed chamber which is subject to progressive decompression (11). As atmospheric pressure falls, there is a proportional decrease in oxygen partial pressure. This results in hypobaric hypoxia, which causes reduced motor and cognitive capacity and eventual loss of consciousness and death. Recent comprehensive investigations in poultry have concluded that LAPS is humane in broiler chickens, with a welfare impact equivalent to CAS with inert gases (12–15). In light of this, in 2018 LAPS was added to the EU regulatory framework on the protection of animals at the time of killing as an amendment [EU Directive 2018/723, amendment to 1099/2009 (16)] as an approved method for stunning of broilers up to 4 kg and for depopulation purposes.

In a recent review of the potential merits and feasibility of LAPS in pigs commissioned by the UK Agriculture and Horticulture Development Board for Pork (AHDB Pork), Bouwsema and Lines (9) concluded that LAPS has the theoretical potential to provide an improved stunning approach for pigs compared to CO_2_, whereby a system based on multiple chambers could allow group stunning and minimal stressful handling. They calculated possible cycle times and throughput rates and suggested, based on ascent rates that are known to be unproblematic for humans, that an equivalent ascent from 0–13,716 m over 5 min (averaging 45.7 ms^−1^) may be a suitable starting point for further investigations. However, this ascent rate followed by a proposed dwell time of 7 min would result in a lengthy 16-min cycle duration, which may not be commercially viable. If the LAPS cycle applied to poultry was used, then a target altitude of 11,498 m could be reached in ~124 s (averaging a decompression rate of 127 ms^−1^, with minor fluctuations dependent on ambient environmental parameters (e.g., elevation, temperature and relative humidity) (11, 12), which along with dwell time and loading/unloading, could support a more realistic cycle time of ~9 min (9). However, there is a lack of data on the responses of pigs to decompression in these ranges, and care must be taken when extrapolating to pigs from poultry given their significant physiological and anatomical differences. Only a single study is published where hypobaric hypoxia was used as a killing method in pigs, reporting research undertaken by the USA National Pork Board (17), as well as an MSc thesis (18). Their focus was on exploring hypobaric hypoxia as a potential on-farm euthanasia method, and they exposed pre-weaned piglets to hypobaric hypoxia with an average ascent rate of 39.6 ms^−1^ (with a peak altitude of 18,000 m over 27.4 ± 6.7 min). Basic behavioral, physiological and pathological data were collected and while effective in causing death, post-mortem examination revealed that 20.7% of exposed piglets displayed lesions including small air bubbles in the epidermis, subcutaneous tissues and fat as well as lung lesions including pulmonary oedema and pleural petechiae. These pathological findings may relate to decompression sickness (possible with the extended exposure times used) or, more likely, relate to the extremely high-altitude equivalent conditions achieved which are near Armstrong's line (and not necessary for hypoxic death). While useful to confirm that hypobaric hypoxia causes gradual loss of consciousness and death in piglets, these findings have limited value when predicting responses to different decompression cycles and to larger pigs.

Pathological outcomes related to decompression exposure provide important insights into possible welfare harms, and also the viability of LAPS in meat production, since it is crucial that any new slaughter method allows product quality to be maintained. Due to the mode of action of hypobaric hypoxia, there are concerns that injuries may be caused as a result of gas expansion in body cavities as a result of the decompression process, which could be painful and distressing to a conscious animal. Expansion of air in the respiratory system, alimentary tract, sinuses of the skull, teeth and middle ear are the most likely sources of potentially painful sensations during gradual decompression, and in pigs, some of these tissues (e.g., lungs) are collected as part of the pluck and have economic value as offal (19). Additionally, pigs are susceptible to lung lesions and respiratory problems (10, 20), which may result in pain and distress during decompression as well as influence their responses to hypoxia.

Encouragingly, studies in poultry have demonstrated that LAPS has no detrimental impact on meat quality (21–23) and that there is no compromise to organ integrity (15), but these effects are likely to be species specific and the rate of decompression as well as the final vacuum pressure is crucial for mitigating pathological and clinical issues (24–26). In humans, slow rates of decompression may be associated with clinical signs such as tooth and middle ear pain, abdominal discomfort and joint pain but these are more common with descent of aircraft than ascent (27–30). Commercial LAPS for poultry achieves an absolute vacuum pressure of ~20 kPa, which means ebullism is highly unlikely to occur (11, 31), and it should be noted that in birds, gases are unlikely to be trapped in the lungs or abdomen during LAPS because of the unique anatomic structure of the avian respiratory system (32). In mammals, expansion of air in the lungs may be more problematic, requiring a slower rate of decompression to allow pressure equalization.

The aim of this study was to characterize pathological changes in pigs resulting from exposure to a range of candidate decompression profiles for non-recovery stunning. To protect welfare, we worked on unconscious, terminally anesthetized pigs, with the objective of identifying a potentially suitable decompression rate to be further investigated in conscious animals. As part of a systematic investigation of whether hypobaric hypoxia could be the basis of a humane, reliable and efficient method of stunning for commercial pigs, we investigated four decompression rates, all achieving the same final pressure. This study is Part 2 of a pair, with behavioral and physiological findings from the same experiments published in Part 1 (33).

## Methods and materials

### Ethical approval

This study was conducted at the University of Edinburgh, following ethical approval from both the University of Edinburgh and SRUC Animal Welfare and Ethical Review Bodies (AWERBs, study approval refs: L325 and ED AE14-2018) and project license approval from the Home Office (PPL: PF5151DAF; Protocol 3). All work is reported to be fully compliant with the ARRIVE guidance. Daily monitoring of all animals was performed and no adverse effects were reported.

### Animals, housing and husbandry

Sixty 10-week old weaner-grower Large White (LW) x Landrace (LR) x Danish Duroc (DD) pigs (Rattlerow Farms Ltd, Suffolk, UK), balanced for sex and weighing ~30 Kg (mean = 29.6 ± 0.5 Kg) were sourced from SRUC's pig unit and moved to the University of Edinburgh's research facility. All pigs were healthy and assessed as fit to travel before being recruited into the trial. Pigs were moved in familiar groups to reduce distress and aggression. On arrival, the pigs were housed in groups of six per pen in large pens [4 × 4.6 m (18.4 m^2^)] bedded with deep straw and wood shavings, in climate-controlled rooms and lights on a timer (06:00–18:00). Pigs were provided with *ad libitum* access to water through adjustable height drinkers and dry pelleted feed (Ultra G200, ForFarmers, UK). Pens were supplemented with large dog chew toys to provide additional enrichment. Following transportation, pigs were given 48 h minimum to acclimatize to their new surroundings prior to experimental work starting.

### Pre-stun anesthesia procedures and physiological monitoring

The pigs were anesthetized for the stun process which was maintained intravenously. Twelve hours prior to anesthesia food was withdrawn from the group in order to prevent complications with anesthesia. On experimentally assigned stun days, pigs were gently moved in pairs into the anesthesiology room and housed in a pen according to treatment order where they were sedated. These pens (1.2 × 1 m) had rubber matting on the floor (supplemented with straw) and semi-solid walls to prevent touching/interference from neighboring pigs in adjacent pens, but visual and olfactory contact was maintained.

Sedation was induced with azaperone 1 mgkg^−1^, ketamine 5 mgkg^−1^, midazolam 0.25 mgkg^−1^ and medetomidine 10 μgkg^−1^, combined in one syringe and administered *via* intramuscular injection to the brachiocephalicus muscle in the neck. Sedation occurred within an average of 15.3 ± 0.8 mins (range 12–31 mins). Sedated pigs were lifted onto a table and, if required, isoflurane vaporized in oxygen (minimum F_I_O_2_ 0.3) and nitrous oxide was administered *via* face mask. An auricular vein was cannulated, after which anesthesia was maintained intravenously with an infusion of propofol at 0.2 mgkg^−1^ minute^−1^. The trachea was intubated and the pigs spontaneously breathed oxygen *via* a Bain breathing system whilst physiological monitoring instrumentation was put in place. A multiparameter anesthesia monitor [Datex Ohmeda (GE) S/5 Compact Anesthesia Monitor, US] was used to monitor a number of physiological variables [e.g., heart rate, respiration rate, and peripheral capillary oxygen saturation (SpO_2_)]. Disposable adhesive press-stud electrode sensors (Ambu Blue Sensor M-00-S/50, Ambu, UK) were applied to the pig's limbs and secured with adhesive tape allowing electrocardiogram (ECG) recording and a pulse oximeter probe was clipped to the ear, allowing for detection of SpO_2_. Respiration was monitored by sidestream sampling of CO_2_ via a connector attached to the proximal end of the endotracheal tube. An adhesive bispectral index sensor (BIS, BIS™ Quatro sensors Aspect Medical Systems, USA) was placed on the head and connected to a BIS™ Complete 2-Channel Monitor (Medtronic, USA). The BIS sensor was further secured by a conforming bandage. Both the anesthetic and BIS monitor allowed continuous monitoring of the pig's physiological variables and evaluation of anesthetic depth. Additionally, disposable adhesive press-stud electrode sensors (Ambu Blue Sensor M-00-S/50, Ambu, UK) were placed on the thorax and connected to a custom-made battery-powered telemetry/logging device, housing a micro-SD memory cards (SanDisk 32GB, Maplin Electronics Ltd. Rotherham, UK), allowing continuous data logging of ECG waveforms at a sampling rate of 1,000 Hz (34). All pigs had an additional auricular venous cannula inserted in order to allow for rapid administration of substances (i.e., overdose of barbiturates) for emergency euthanasia if required.

### Gradual decompression and the LAPS^®^ system

The LAPS^®^ system was developed by TechnoCatch LLC, USA for the stunning of poultry (11). Briefly, the system utilizes a large cylindrical chamber, with bespoke monitoring and control systems designed to operate desired decompression cycles. There are multiple sizes of chambers available as part of the LAPS^®^ system, all operating in the same way, but allowing for specific uses. In this study we used a chamber developed for research purposes (2.5 m diameter, 3.7 m long), which allowed for an automated programmable logic controller (PLC) system, providing flexibility in decompression rate settings. The PLC recorded the chamber pressure (mmHg), temperature (°F), relative humidity (%) and atmospheric oxygen (%) at the start and during each executed cycle. The chamber had an automated hydraulic door, operated from the central PLC. Decompression cycles were pre-programmed in order to achieve target decompression rates selected, but followed the same overall cycle profile as the commercial poultry settings, with two phases (11, 12). Phase 1 involved the vacuum chamber pressure being reduced from atmospheric pressure to an absolute vacuum of ~33 kPa (equivalent to 8,459 m); and the second phase (hold phase) involved modulation by a sliding gate valve, reducing the pumping speed *via* “choke flow” and slowing the decompression in the chamber to the final absolute vacuum of ~20 kPa (equivalent to 11,498 m). The length of each phase was dependent on decompression rate selected for each treatment; however, the total cycle length was fixed to 720 s (12 mins) or 480 s (8 mins). The reduction in total pressure causes a synchronized reduction in oxygen partial pressure, and therefore a reduction in oxygen available to breathe. At the end of the cycle, the chamber is returned to atmospheric pressure over a fixed period of 60 s of recompression using a baffled air inlet. The LAPS^®^ system was housed within a large barn with direct access to animal and anesthesia facilities, with the study site located at ~67 m altitude (absolute atmospheric pressure at ~101 kPa).

The decompression chamber was modified to allow for several additional sealable ports to be placed, allowing for additional cabling to be run through and power equipment within the chamber, without compromising vacuum pressure. The chamber was lit by two dimmable LED lighting strips (RS PRO White LED Strip, RS Components, UK), set to 180 lux and positioned to the left and right of the central line on the ceiling. Two temperature and relative humidity loggers (Tinytag Ultra 2, TGU-4500, Gemini Data Loggers, UK) were placed at pig level and set to record data at 10 s intervals. To record and monitor both pig behavior and anesthetic monitors (within the chamber), two Ezcctv GeoVision surveillance systems (GV1480 - 16 camera video capture card, ezCCTV, UK) were installed outside the chamber and connected through the sealable ports to multiple CCTV cameras, secured in multiple locations by a custom-built camera rig supplemented with adjustable arms and clamps (Manfrotto, UK). The first system monitored and logged the behavior of each pig with individual cameras from a frontal (facial) angle (Sony Gamet Effio, SpyCameraCCTV, UK) and aerial cameras (CCD Bird Box Camera, SpyCameraCCTV, UK). The second system involved individual cameras (Bullet LED, SpyCameraCCTV, UK) focused on each anesthesia and BIS monitors within the chamber. The surveillance systems provided not only recorded footage for later in-depth analysis, but also live footage of the pigs and the anesthetic monitors to three desktop monitors (Dell, UK) outside of the chamber, allowing for continuous and immediate assessment of each pig pre-, during and post treatment cycle. Furthermore, two external dynamic microphones (Shure, UK) were fitted inside the chamber at pig height, ~50 cm in front of each pig. The microphones were connected to a portable audio recorder (Tascam DR 100-MKII Linear PCM recorder, Tascam, USA).

Based on previous decompression studies in poultry (11–15, 21–23) and mammals (including humans) (17, 18, 29, 35) we initially selected three target decompression rates to apply to pigs: 60 ms^−1^, 80 ms^−1^ and 100ms^−1^ over a total cycle time of 720 s (12 mins), which we hypothesized would cause the pigs to enter a non-recovery state with minimal pathological consequences. Crucially, these rates also matched hypothesized feasible cycle times for commercial slaughter of pigs (9). The study was flexibly designed to allow the inclusion of additional refinement curves, based on preliminary findings as the study progressed. Decompression cycles were applied to all 60 pigs, in pairs, across 6 days in total, with the first 3 days including only the initial pre-selected decompression rates. Following this, preliminary analyses were conducted (3 cycle pairs of pigs per treatment) and subsequently two additional “refinement” curves were added to the experiment, which included a slower rate of 40 ms^−1^ (cycle time = 720 s) and a matched rate of 60 ms^−1^, but with a reduced cycle time of 480 s, to examine whether a reduced ‘hold' period at low pressure was related to pathological outcomes.

### Experimental procedure

Male and female pigs were randomly assigned into mixed pairs, blocked by home pen, in order to ensure familiarity and reduce stress associated with individual housing (36). Pairs were initially randomly assigned to one of three decompression treatments according to a randomized-block factorial design using a Latin square. Assignment was blocked by home pen [to prevent distress with single pairs of pigs being left overnight in home pens (36)]. However, following the inclusion of the additional two refinement decompression treatments, the remaining pigs (42 pigs), were re-assigned according to a second randomized-block factorial design using a Latin square to one of five decompression treatments, blocked by home pen and partially by day. The experiment took place over 6 days, with 6 pigs (3 cycles) being exposed for the first 2 days, and 12 pigs (6 cycles) exposed per day for the remaining 4 days. As a result, a total of 12 pigs (6 pairs) were exposed to one of five decompression treatments.

Following the completion of anesthesia monitoring instrumentation, each anesthetized pig was placed in an adapted dog surgical sling, designed with four leg openings. The pig (within the sling) was then carefully lifted into a handling crate (L:1500 × W:1007 × H:800cm) equipped with struts, allowing the pig within the sling to be suspended in an upright position. The crate was custom built and consisted of a galvanized steel frame with clear polycarbonate sides and doors - enabling pairs of pigs to be safely housed in individual compartments during stunning with an unobstructed view for the closed-circuit television (CCTV) cameras within the chamber. A shelf was fitted to the back of the crate and housed the anesthesia and BIS monitors.

Once pairs of anesthetized pigs were placed in the crate, it was immediately maneuvered *via* forklift truck to the decompression chamber and carefully positioned to ensure clear camera views of the pigs and the monitors. Individual pig propofol infusion was maintained during transport *via* fluid lines. During decompression, these were passed through modified sealable ports of the chamber and running through installed ceiling rails, which allowed infusion pump control from outside the chamber, but without compromising the internal vacuum. At all times during the decompression cycle pigs were continuously administered propofol intravenously at 0.2 mgkg^−1^ minute^−1^. A lethal dose of pentobarbital (80 mgkg^−1^) was connected to the fluid lines *via* a three-way stopcock for use in the event that immediate euthanasia became necessary.

The chamber door was then closed, and a 30 s baseline recording for physiological and behavioral measures commenced. The decompression treatment was then applied, according to design allocation. During the cycle, the live footage of the anesthetic monitors was constantly observed by a veterinary anesthetist and a senior scientist who monitored anesthetic depth and confirmed timings of cardiac arrest (based on loss of pulse), cessation of breathing and brain death (as indicated by BIS) in real time. Trained staff provided real-time monitoring of decompression cycle parameters as indicated by the PLC output. Following confirmation of cardiac arrest and brain death, the cycle was terminated at the prescribed cycle length (e.g., 720 or 480 s, according to treatment) and the chamber was immediately recompressed at a fixed rate over 60 s and the door was opened. The crate was removed *via* forklift and death confirmed by a veterinarian based on absence of heart sounds (on auscultation) and cranial nerve reflexes. The pigs were removed from the crate and the sensors, loggers and intravenous cannulae were removed.

### Pathological assessment

#### Preliminary pathological examination

To obtain an accurate baseline, all pigs underwent pre-treatment ante-mortem external examinations in the home pen ~30 mins before sedation. Binary (yes/no) recordings of external injuries, including abrasions, lesions, bruising and discharge were recorded for the eyes, ears, nose, oral cavity (including the tongue), external orifices (including anus and vulva) and whole-body. Immediately after decompression treatment and removal from the chamber, identical observations were recorded post-mortem, noting any new injuries which had occurred. The same trained observer recorded all ante- and post-mortem preliminary evaluations.

#### Detailed pathological examination

A pseudo-randomly assigned subset of the pigs were submitted for a full detailed post-mortem pathological examination at SRUC Veterinary Services, with a total of 6 pigs per treatment, balanced for sex. Sample size estimation was conducted using Bayesian power analysis *via* R software version 4.1.3 [R Core Team (v. 2022): R: A Language and Environment for Statistical Computing], through R Studio (2022.02.1 Build 461, RStudio, PBC, 2009-2022) and packages [simstudy (37)]; [bayesplot (38)]; and [posterior (39)]. Simulations were based on binary pathology data from pilot studies in pigs (18) and other species (15) exposed to decompression killing. The projected sample size needed was *N* = 6 per treatment. All post-mortem examinations were performed by the same team of two people, including a specialist porcine veterinary pathologist (JT) and a post-mortem assistant (CW), both blinded to treatment. Post-mortem examinations occurred the same day as the stunning treatment and on average within 4.2 ± 0.3 h of exiting the chamber (min = 49 mins; max = 6h 59mins). All findings were recorded on a pro-forma detailing features such as congestion, hemorrhage and any other abnormalities in all specified carcass sites and organs. The full examination included external examination of the carcase, the ears, oral cavity including the gingiva, tongue, teeth and pharyngeal tissues, the conjunctivae and externally-visible tissues of the eyes most notably the sclera, the nasal planum and nostrils, other external orifices (anus and vulva), the nasal cavities including the nasal conchae, the frontal sinuses, cranium, meninges, brain, muscles and connective tissues of the neck, esophagus, larynx, trachea, lungs, pericardium, heart, thoracic and abdominal cavities, liver, spleen, kidneys, stomach, duodenum, pancreas, jejunum, ileum, cacum, colon, colon load, rectum and representative joint cavities in the limbs (stifles and elbows). For each site, congestion and/or hemorrhage were scored using an ordinal scale of 0 to 5 to indicate degree of change from what would normally be observed. Scoring categories were defined as: 0 = no change; 1 = a very slight but noticeable change; 2 = a low grade change; 3 = a moderate change; 4 = a marked change; and 5 = a severe change. At the veterinary pathologist's discretion, photographs were taken of specific sites.

### Statistical analyses and data processing

Statistical analysis was performed using R software version 4.1.3 [R Core Team (v. 2022): R: A Language and Environment for Statistical Computing], through R Studio (2022.02.1 Build 461, RStudio, PBC, 2009-2022). Data were processed and tidied using the tidyverse package (40). First comparisons involved modeling for differences between rates with the total cycle time of 720 s (40, 60, 80, and 100 ms^−1^). Secondary comparisons explored differences between cycle length within the single decompression rate of 60 ms^−1^.

Cumulative Link Mixed Models (CLMMs) were used for post-mortem parameter response variables [packages: ordinal (41) and RVAideMemoire (42)] to compare congestion and hemorrhage scores (0–5) in organs and tissues with the threshold set to equidistant. Model fitness was verified using the DHARMa package (43) and nominal and scale test functions in the ordinal package (41). All models included pair as a random effect. Final model selection followed the “top-down” method (44), but all final models included decompression rate (or cycle duration) and sex as fixed effects, and temperature, pig weight, relative humidity and time to post-mortem following cycle end as co-variates. We assessed significance of explanatory variables with the ANOVA function in the car package (45) and estimated pairwise comparisons with the emmeans function with mode set to “mean.class” to obtain the average probability distributions as probabilities of the scores 0–5 and “prob” to obtain estimates of the probability distribution of each rating (46), using tukey adjustment of the *p* values to account for multiplicity. Where feasible, all body regions were modeled to explore fixed effects, however in some cases modeling was not possible due to little or no variation in pathological scores.

Binary post-mortem measures were modeled using generalized linear mixed models with the family link set to “binomial” using the function glmer in the package lme4 (47). Model fitness was verified using the DHARMa package (43). Similarly, to the CLMMs, we included pair as a random effect and all final models included decompression rate (or cycle duration) and sex as fixed effects, and temperature, pig weight, relative humidity and time to post-mortem following cycle end as co-variates. As described above, we assessed significance of fixed effects using the ANOVA function (45) and estimated pairwise comparisons with the emmeans function (46), using Tukey adjustment of the *p* values to account for multiplicity. Graphical summaries were produced using the corrected pairwise comparisons using the ggplot2 package (48). Unless stated, we found no effects of sex, crate side or co-variates weight, time to post-mortem or environmental parameters.

## Results

All pigs were killed as a result of exposure to all decompression cycles, irrespective of rate or length, and none required emergency euthanasia. Physiological real-time monitoring confirmed that no pig regained consciousness following pre-cycle sedation and anesthesia or during the decompression cycles (33). Details relating to decompression cycle profiles (e.g., decompression rate ranges) and environmental factors (e.g., ambient temperature and relative humidity) are reported and published in Part 1 (33). In brief, exact target decompression cycle rate averages were difficult to replicate in a non-environmentally controlled outdoor facility. Unlike the commercially available LAPS system for poultry, where adaptations to ambient temperature and relative humidity are automated (11), the experimental unit used in this study was manually programmed for each cycle. This resulted in achieved decompression rates being generally lower than desired (mean (±95% CI) decompression rates per target cycle: 40ms^−1^ = 42.27 ms^−1^ (41.19, 43.36 ms^−1^); 60 ms^−1^ (combined 720 and 480 s cycle lengths) = 54.09 ms^−1^ (51.66, 56.52 ms^−1^); 80 ms^−1^ = 74.75 ms^−1^ (70.31, 79.19 ms^−1^); 100 ms^−1^ = 89.84 ms^−1^ (88.45, 91.23 ms^−1^). Ambient temperature and relative humidity, as expected fluctuated across experimental days and within cycle periods.

There were two unexpected incidents during two decompression cycles, both caused by a malfunction with the anesthesia equipment (syringe driver malfunction) external to the chamber, which resulted in temporary aspiration of air and rapid administration of propofol to 3 pigs (cycle run 16 (80 ms^−1^) both pigs and cycle run 28 (80 ms^−1^) single pig. These occurred late in the cycle therefore the physiology and behavior data up to the incidents were included but these animals were excluded for pathology.

Real-time monitoring of anesthesia monitors provided a conservative estimate of latency to cardiac arrest and was defined by permanent loss of mechanical cardiac activity, represented by the time at which the pulse pressure contour on the pulse plethysmograph became permanently imperceptible by the supervising veterinary anesthetist. Mean latencies (±SE and 95% CI) to cardiac arrest ranged from 120 to 205 s (40 ms^−1^ = 204.0±24.3 s (153.4, 254.0 s); 60 ms^−1^ (combined 720 and 480 s cycle lengths) = 191.0 ± 16.3 s (156.9, 225.0 s); 80 ms^−1^ = 173.0 ± 23.3 s (123.5, 222.0 s); and 100 ms^−1^ = 128.0 ± 22.7 s (80.2, 175.0).

### Preliminary pathological examination

All pigs showed no sign of injury during pre-treatment preliminary examination and ante-mortem inspections. In the longer cycles (720 s), post-mortem external examination revealed that some pigs, irrespective of decompression rate showed lesions to both sides of their body, likely as a result of contact with the holding crate during convulsive activity. This included minor bruising and abrasions to the skin (Table 1), but there was no difference in the probability of pigs showing these injuries between decompression rates, suggesting convulsive activity was similar across rates. Similar results were seen in the shorter decompression cycle when comparing the two 60 ms^−1^ decompression rate groups only, with no differences as a result of cycle length (left body lesions: $X_{\left(1,23\right)}^{2}$ = 2.19, *p* = 0.1388; right body lesions: $X_{\left(1,23\right)}^{2}$ = 0.02, *p* = 0.9905). A proportion (>25%) of pigs across all decompression rates were observed to have congested (and in some cases haemorrhagic) sclera post-treatment, with an indication of higher proportions of pigs exhibiting this in the faster decompression rates (e.g., 80 and 100 ms^−1^), however comparisons were not significant for the left or right eyes dependent on cycle rate (Table 1) or cycle length within the 60 ms^−1^ decompression rate (left eye: $X_{\left(1,23\right)}^{2}$ = 0.60, *p* = 0.4359; right eye: $X_{\left(1,23\right)}^{2}$ = 0.00, *p* = 0.9999).

**Table 1:** Preliminary pathological examinations for the presence (N^+^) of body lesions associated with external trauma/injury, congestion and/or hemorrhage of the sclera (bloodshot eyes), and where applicable statistical comparisons between target decompression rates for 720 s cycles.

**Preliminary pathological assessment parameters**	**Target decompression rate (ms^−1^)**	**N**	**N^+^**	**Mean probability of injury (95% CI)**	***X* ^2^**	***P* value**
Body lesions (left)	40	12	2	0.15 (0.04, 0.46)	2.43	0.4883
	60	12	4	0.32 (0.11, 0.62)		
	80	12	1	0.07 (0.01, 0.39)		
	100	12	3	0.24 (0.07, 0.55)		
Body lesions (right)	40	12	0	0.00 (0.00, 1.00)	2.56	0.5211
	60	12	2	0.17 (0.04, 0.48)		
	80	12	1	0.08 (0.01, 0.41)		
	100	12	4	0.33 (0.13, 0.62)		
Bloodshot eye (left)	40	12	6	0.50 (0.20, 0.80)	1.86	0.6018
	60	12	3	0.22 (0.05, 0.59)		
	80	12	6	0.50 (0.20, 0.80)		
	100	12	6	0.50 (0.20, 0.80)		
Bloodshot eye (right)	40	12	5	0.40 (0.13, 0.74)	5.2	0.1575
	60	12	3	0.21 (0.05, 0.60)		
	80	12	9	0.78 (0.40, 0.95)		
	100	12	9	0.78 (0.40, 0.95)		

No pigs were observed to have injury to their teeth, ears, external orifices or have discharge from their mouths or ears pre and post-treatment. A single pig (subjected to the 40 ms^−1^ rate) had a minor injury to its tongue, but no other tongue injuries were observed. A total of five pigs had minor nasal discharge which was observed at the faster decompression rates (1 pig at 60 ms^−1^ (480 s cycle), 2 pigs for both 80 and 100 ms^−1^ decompression treatments), however due to so few observations, statistical modeling to compare differences across treatments was not possible. The nasal discharge of one pig contained blood (80 ms^−1^ rate), with all other nasal discharge reported as clear nasal mucus. Two pigs presented with rectal prolapses and were those exposed to 40 and 100 ms^−1^ treatments and therefore both longer cycle lengths (720 s).

### Detailed pathological examination

The counts of pigs observed to have each pathological score (0–5) are reported in Supplementary Table S1 (total of 30 pigs) for cranial regions and Supplementary Table S2 for the more caudal regions and extremities. Exposing pigs to decompression and the unavoidable subsequent recompression resulted in generalized congestion of the carcase, organs and body cavities including the ears, oral cavity, conjunctivae and sclera, mucosa of other external orifices (anus and vulva), nasal planum, nasal cavities including nasal conchae, frontal sinuses, cranium, meninges, brain, larynx, trachea, lungs, heart, parietal pleura of the thoracic cavity, peritoneum of the abdominal cavity, stomach, small intestine, caecum, colon, liver, spleen and kidneys and representative joint cavities in the limbs (stifles and elbows). Marked and/or severe congestion (scores 4 and 5) was seen in the conjunctivae and sclera, frontal sinus, nasal cavities including nasal conchae, cranium, meninges, brain, lungs, liver, spleen, kidneys, intestines and mucosa of other external orifices (anus and vulva). No hemorrhage was observed in the ears, oral cavity, tongue, teeth, nasal planum, heart, abdominal cavity, stomach, duodenum, small intestine, pancreas, caecum or colon, irrespective of target decompression cycle. Various severities of hemorrhage were observed in the conjunctivae and sclera, mucosa of other external orifices (anus and vulva), nasal cavities including nasal conchae, frontal sinuses, cranium, meninges, brain, larynx, tracheal lumen, lungs, parietal pleura of the cranial and caudal thoracic cavity, liver, spleen and kidneys and representative joint cavities in the limbs (stifles and elbows). The most severe scores were seen in the conjunctiva and sclera at the fastest target rate (100 ms^−1^) and in the slower rates for both lungs (40 and 60ms^−1^ target rates). Reviewing both congestion and hemorrhage scores, only the teeth, pancreas and carcase tissues in the cervical region showed no signs of impact as a result of decompression or recompression.

**Figure 1:**
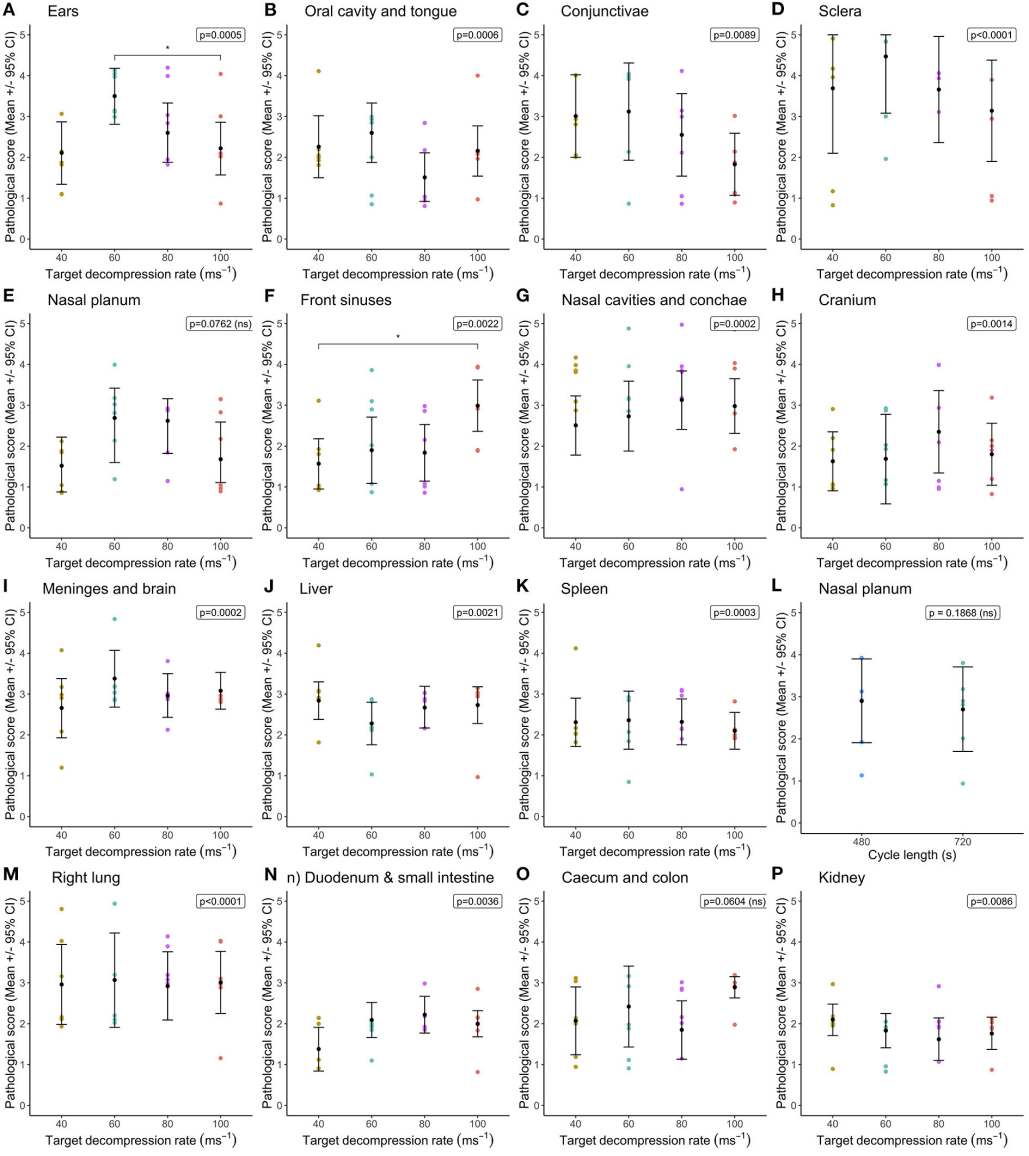
Comparison of mean [±95% confidence intervals (CI)] congestion scores across decompression rates (40, 60, 80, and 100 ms^−1^) within the longer cycle length (720 s) for multiple organ and tissue sites, where variation in scores permitted modeling. Including: **(A)** ears; **(B)** oral cavity and tongue; **(C)** conjunctivae; **(D)** sclera; **(E)** nasal planum; **(F)** frontal sinuses; **(G)** nasal cavities and conchae; **(H)** cranium; **(I)** meninges and brain; **(J)** liver; **(K)** spleen; **(L)** left lung; **(M)** right lung; **(N)** duodenum and small intestine; **(O)** caecum and colon; and **(P)** kidney. The ordinal scale represented the degree of change from what would normally be observed (0 = no change; 1 = a very slight but noticeable change; 2 = a low-grade change; 3 = a moderate change; 4 = a marked change; and 5 = a severe change).

**Figure 2:**
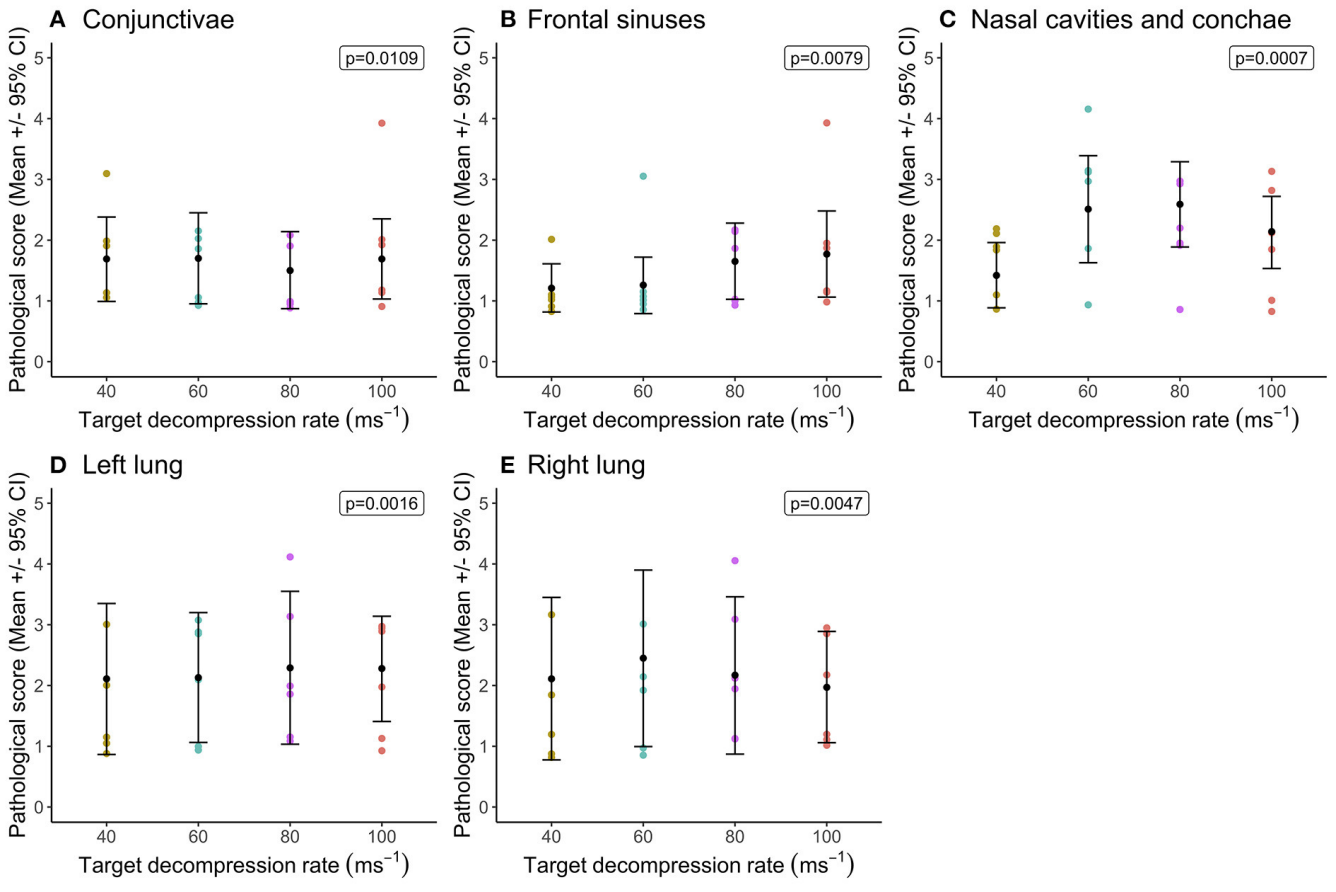
Comparison of mean [±95% confidence intervals (CI)] hemorrhage scores across decompression rates (40, 60, 80, and 100 ms^−1^) within the longer cycle length (720 s) for key organ and tissue sites, where variation in scores permitted modeling. Including: **(A)** conjunctivae and sclera; **(B)** fontal sinuses; **(C)** nasal cavities and conchae; **(D)** left lung; and **(E)** right lung. The ordinal scale represented the degree of change from what would normally be observed (0 = no change; 1 = a very slight but noticeable change; 2 = a low-grade change; 3 = a moderate change; 4 = a marked change; and 5 = a severe change).

Statistical comparisons between decompression rates (40, 60, 80, and 100 ms^−1^) within the longer cycle length (720 s) highlighted widespread differences in congestion scores, however very few pairwise differences were identified despite overall effects of decompression rate (Figure 1). In general, faster decompression rates produced higher scores in the frontal sinuses, nasal cavities and conchae, cranium, meninges, brain, left lung, duodenum and small intestine. However, faster decompression rates were associated with marginally lower congestion scores in the conjunctivae, sclera and kidneys. In some body regions, decompression rates were shown to have an overall effect on congestion scores, but the magnitude of the differences was minimal. The front sinus was the single area which showed a marked increase in congestion score with decompression rate (X${}_{\left(3,23\right)}^{2}$ = 14.63, *p* = 0.0022), as well as pairwise comparisons revealing a difference between 40 and 100 ms^−1^ (Figure 1F). There was considerable individual variation in pathological scores across all body regions, which could not be explained by the fixed effects or co-variates included within the models. This is illustrated in variation in hemorrhage scores, albeit with a general trend for the nasal cavities including the conchae and frontal sinuses to have higher scores for pigs exposed to higher decompression rates (Figure 2).

All pigs had serosanguineous fluid in the abdomen (mean = 28.9 ± 5.6 ml (95% CI = 21.3, 49.4 ml) and in the pericardial cavity (mean = 10.6 ± 2.1 ml (95% CI = 6.0, 15.2 ml), but the volume of fluid was not affected by decompression rate. Some pigs (*n* = 6) exhibited foam from the nose and mouth post decompression/recompression, irrespective of decompression rate.

**Figure 3:**
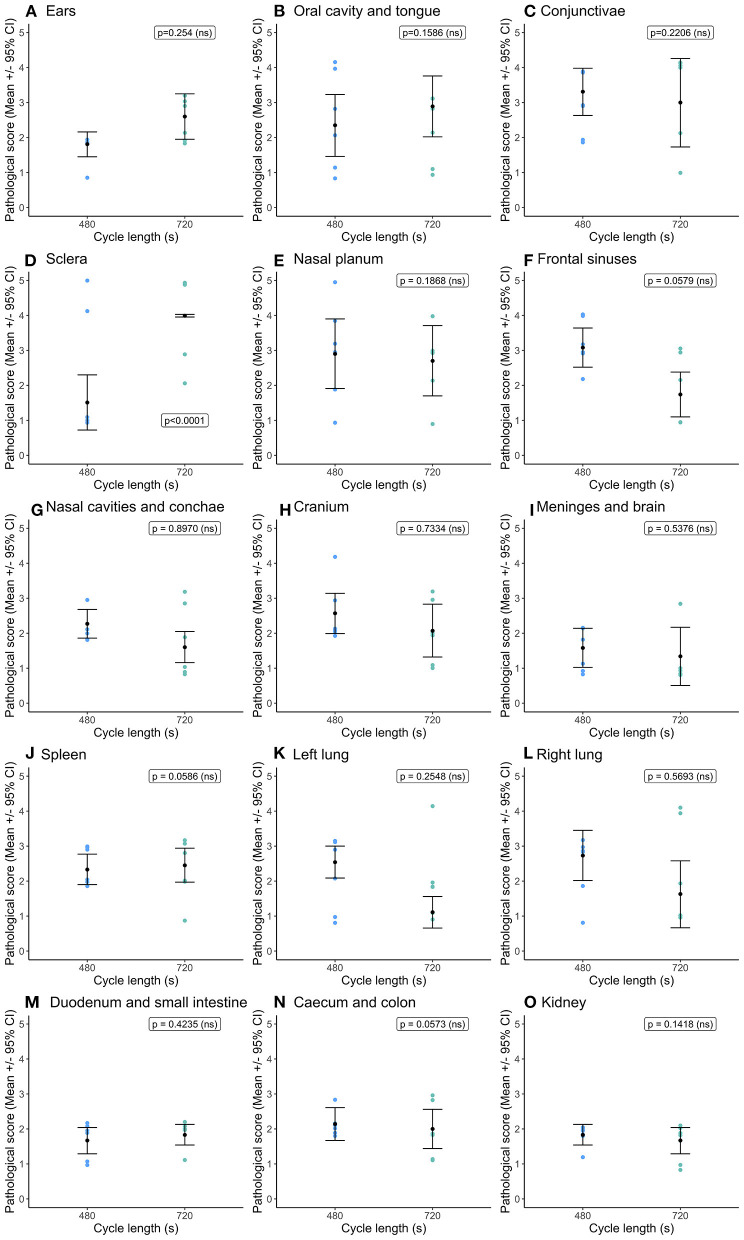
Comparison of mean [±95% confidence intervals (CI)] congestion scores for key organ and tissue sites, where variation in scores permitted modeling dependent on cycle length (480 vs. 720s) for the single decompression rate of 60 ms^−1^. Including: **(A)** ears; **(B)** oral cavity and tongue; (C) conjunctivae; **(D)** sclera; **(E)** nasal cavities and conchae; **(F)** frontal sinuses; **(G)** nasal cavities and conchae; **(H)** cranium; **(I)** meninges and brain; **(J)** spleen; **(K)** left lung; **(L)** right lung; **(M)** duodenum and small intestine; **(N)** caecum and colon; and **(O)** kidney. The ordinal scale represented the degree of change from what would normally be observed (0 = no change; 1 = a very slight but noticeable change; 2 = a low-grade change; 3 = a moderate change; 4 = a marked change; and 5 = a severe change).

Overall, there were no differences in congestion scores (Figure 3) between cycle lengths within the 60 ms^−1^ decompression target rate. The only difference observed was in the conjunctivae and sclera (Figure 3D), where congestion scores were significantly higher in pigs exposed to the longer cycle. As before, there was substantial variation in congestion scores within treatment.

**Figure 4:**
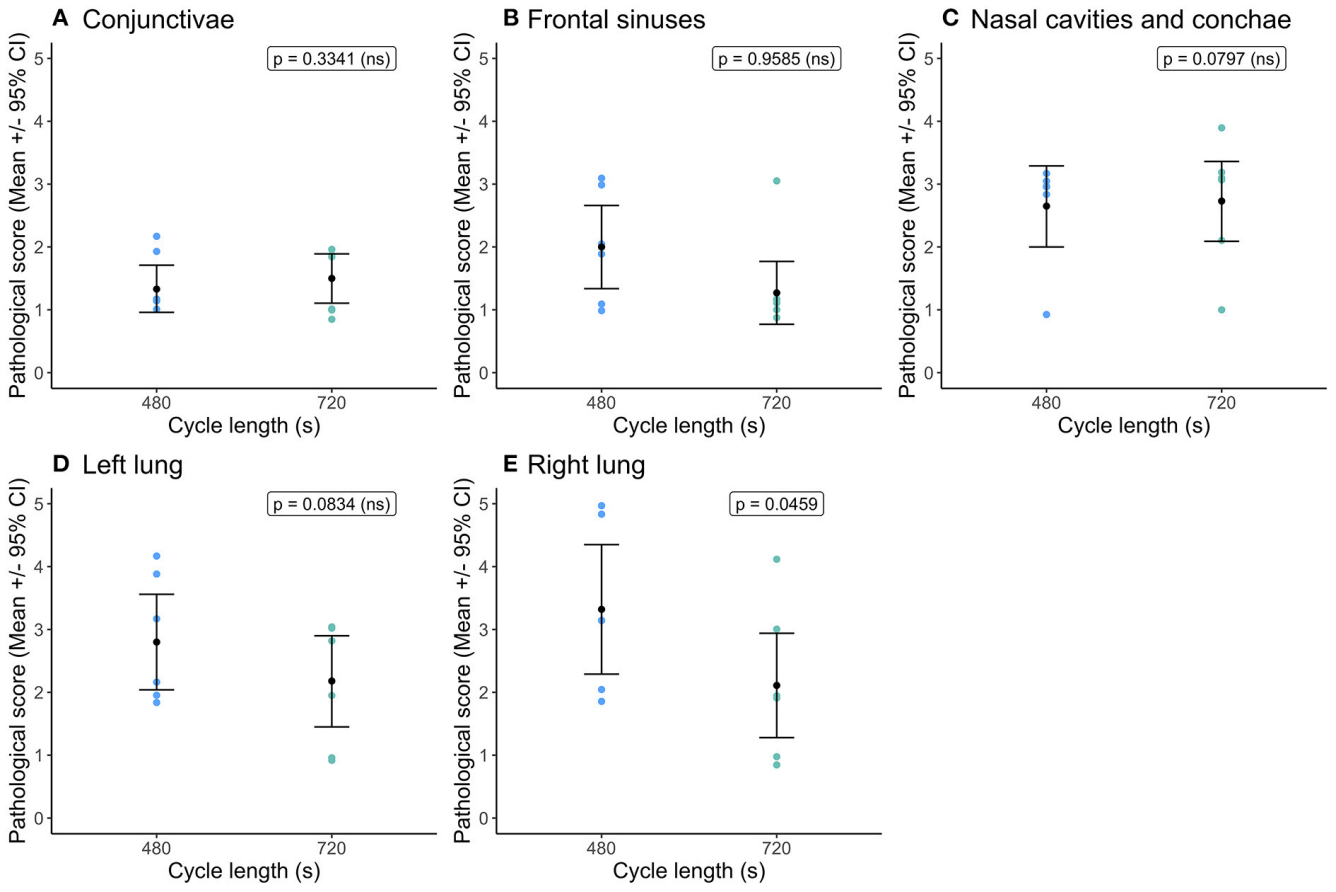
Comparison of mean [±95% confidence intervals (CI)] hemorrhage scores for key organ and tissue sites, where variation in scores permitted modeling dependent on cycle length (480 vs. 720 s) for the single decompression rate of 60 ms^−1^. Including: **(A)** conjunctivae and sclera; **(B)** fontal sinuses; **(C)** nasal cavities and conchae; **(D)** left lung; and **(E)** right lung. The ordinal scale represented the degree of change from what would normally be observed (0 = no change; 1 = a very slight but noticeable change; 2 = a low-grade change; 3 = a moderate change; 4 = a marked change; and 5 = a severe change).

Cycle length had no impact on hemorrhage scores for the conjunctivae and sclera, frontal sinuses, nasal cavities including conchae and left lung (Figure 4). The right lung had higher hemorrhage scores within the shorter cycle length (Figure 4E) and as noted previously sustained the higher severity scores for both congestion and hemorrhage across both cycle lengths. Serosanguineous fluid was present in the abdominal cavity (mean = 26.1 ± 4.9 ml (95% CI = 13.9, 38.2 ml) and pericardial cavity (mean = 10.7 ± 1.4 ml (95% CI = 6.08, 14.9 ml) for all pigs, irrespective of cycle length and no effect on fluid volume. Three pigs in the short cycle and two in the longer cycle exhibited foam from the nose and mouth post decompression treatment (and recompression), however the likelihood of the presence of foam was not significantly affected by cycle length.

## Discussion

Decompression at all target decompression rates and cycle lengths was effective in creating a non-recovery state in all pigs (i.e., all pigs were dead after exposure). Detailed pathological assessments, obtained for the first time in pigs, demonstrated that under certain decompression and subsequent recompression rates internal and external damage (hemorrhage, congestion) in a range of body areas occurs. In some pigs the damage was scored as marked or severe. Whilst this study cannot properly inform the welfare implications (all pigs were anesthetized throughout the procedure and it is not possible to know when in the cycle (i.e., decompression or recompression) the damage is occurring), it is likely that such findings would raise concerns if observed at an abattoir and they certainly have implications for meat quality and condemnation of certain organs. The results here should not be considered in isolation from the behavioral and physiological assessment presented as a companion paper (33).

There are a number of caveats that should be noted when discussing the results and their potential implications. The effects of hypoxia are confounded with effects of hypobaria as the process of decompression includes simultaneous application of both conditions. It is also important to consider the potential impact of the unconscious state of the pigs in this study (a measure taken in order to protect their welfare) when investigating unknown decompression rates and effects of hypobaric hypoxia. Accordingly, welfare implications can only be inferred from the physiological and pathological findings. In addition, unconscious animals are unable to perform active behaviors which may be motivated attempts to equalize pressure differentials e.g., swallowing, chewing, stretching or biting (14, 28, 29, 49–51). The considerable individual variation in pathological scores across all body regions (which could not be explained by the fixed effects or co-variates included within the models) suggests that decompression and associated recompression at the rates applied may result in inconsistent pathological outcomes, but this could also be influenced by the pigs being unconscious. Other caveats are that the pigs were not exsanguinated immediately post-stun, which would be the case in a commercial abattoir. Thus, some findings should be approached with caution. The use of scoring systems based on the proportion of the organ affected is a widely accepted approach in field and experimental studies of pathology. However, it should be recognized that such measures are judgement based and thus subject to observer bias. To minimize this risk, careful definitions were provided by a single very experienced pig pathologist who was responsible for making these assessments.

### Treatment effects

Where variation in pathological scores between treatments was limited, it may relate to generalized effects of decompression, regardless of rate, or possibly due to the relatively long ‘hold' phase at low pressure (~20 kPa) of all treatments, including the shortened 60 ms^−1^ cycle (~300+ s).

A long cycle time was chosen due to the lack of knowledge about the impacts of decompression and subsequent recompression on pigs and an important aim was to protect the pigs' welfare as much as possible whilst undertaking this novel procedure. However, once the project team were confident that the pigs were reaching a terminal state (i.e., no chance of recovery) within the initial cycle time we performed several runs with shorter cycle lengths to ascertain cycle length effects within a single rate. Overall, there were minimal pathological consequences of the shorter cycle. This is important as it aligns the procedure more with food production requirements and makes it more competitive with CO_2_ stunning (9), while still providing ample time for a hold phase to prevent recovery. The long hold time employed during this trial was part of the prudence required to ensure non-recovery. However long hold times at low absolute vacuum and subsequent recompression may also affect pathological outcomes. Rapid recompressions are associated with more trauma to mammals, including humans (27–30). Even though neurophysiological measurements indicated that the pigs were in a non-recoverable state upon recompression further damage to tissue by expediting this process could have negative effects on meat quality.

Described in detail by Martin et al. (33), the target decompression rates investigated in this study represent average decompression rates of phase 1 of the decompression curve profiles. Therefore, pigs are exposed to higher rates of decompression during this phase than the reported average target rates. The LAPS^®^ system (developed by TechnoCatch LLC, USA) used in this study calculates the average decompression rate during set intervals relating to pressure thresholds, generating stepwise programming of the decompression curve (11), with the fastest decompression rates experienced by animals in the first interval (start of cycle (~101 kPa) to the first threshold of ~85 kPa. There is evidence that animal-related outcomes may be related to both the overall average target decompression rate, as well as the range of decompression rates at each stepwise interval to generate the curve profile (52). However, it is important to note that all the target rates investigated here, as well as the regulated commercial broiler LAPS profile (11, 16), are within pressure change ranges experienced by humans as part of altitude flight training, and demonstrate a low prevalence (~9%) of self-reported concerns (e.g., decompression sickness etc.) (53). Although the relevance to the shorter exposure time of the decompression profiles in animal stunning contexts may be limited, with the longer term effects of decompression sickness unlikely to apply (27). Previous studies exploring both explosive and rapid decompression injuries reported no lesions when animals (in this case cats) were decompressed to 35,000 ft (~10,668 m) in 1.5 s, similar to the final equivalent altitude of the decompression curves studied here (equivalent to 11,498 m, but at an exceptionally higher rate of decompression), whilst explosive decompression in rats (< 1 s to 50,000 ft) was characterized by diffuse intrapulmonary hemorrhage (54). The majority of physiological symptoms reported in humans as well as pathology outcomes are associated with descent from altitude (i.e., recompression) rather than ascent to altitude (decompression). A recent study undertaken on military aircrew members decompressed the human subjects from 0 to 25,000 ft in ~4 min, where the subjects were held at altitude for a further 4 min for hypoxia awareness training and then recompressed in two stages: (1) 25,000 to ~18,000 ft in ~2 min and held for a further 10 min prior to stage (2) and final recompression from ~18,000 to 0 ft in ~6 min (55). This study reported low incidences of physiological effects (e.g., physiological reaction, ear blockage, sinus blockage and tooth pain and furthermore were predominantly associated with descent from altitude (i.e., recompression). In this study the recompression of the chamber was ~74 s, and is unlikely to be linear in rate, with higher rates are likely to be experienced. The effects of this rapid recompression may share similarities to the effects of blast injury, where an explosion results in a blast wave which starts with an initial rapid single increase in air pressure, immediately followed by the suction of the blast and associated negative pressure (56, 57). The recompression phase here is likely to be associated with the blast overpressure (increased pressure over atmospheric pressure) of a very mild blast wave given the exposure time and relatively low pressure change (56, 57). Injuries associated with blast overpressure in this studies' context, are likely associated with primary event injuries, which involve the direct interaction of the pressure wave and the body and the resultant multiple organ trauma (56). Examples include conjunctival hemorrhage, tympanic membrane rupture and/or hemorrhage, hemorrhage associated petechiae and ecchymosis of the upper respiratory tract, various severities of hemorrhage of the lungs (i.e., petechiae to pulmonary contusion) and congestion (56). There is also evidence that solid organs are more likely to sustain trauma at higher levels of blast overpressure (58), whilst the organs most frequently injured are hollow viscous organs (e.g., *gastrointestinal tract*), which are associated with hemorrhages (i.e., petechiae to hematomas) and edema (56, 58, 59). However, cessation of cardiac activity several minutes before recompression in this study should have eliminated any haemodynamic effects relating to cardiac function and blood pressure. Critically, a major and unavoidable limitation of this type of work is the separation of effects attributed to recompression and decompression, as recompression is essential following decompression in order to remove and gain access to the exposed animal.

As observed in previous studies, there was no correlation between the weight of the animal and the extent and severity of pathology exhibited (15, 54). However, future work should be undertaken to confirm previous findings and establish the pathology outcomes in slaughter-weight pigs following commercial slaughter processes (e.g., hanging and exsanguination).

### External pathology

Although preliminary pathological examinations of external trauma or injury revealed nothing significantly different between treatments, there were findings worthy of further discussion. These included congested (and in some instances hemorrhagic) conjunctivae and sclera observed in over 25% of pigs as well as a few pigs presenting with sunken eyes, similar to that seen in cases of severe dehydration. As the eyes are an interface between internal and external environments, they are impacted upon by all the changes that occur within the pig and within the LAPS chamber (i.e. internal pressure changes (as body reacts to decompression) and external pressure changes (in chamber during decompression and recompression). Therefore, some degree of damage could be expected. However, pigs showing turgidity or sunken eyes were only from the cycles run at 40 ms^−1^ and 100 ms^−1^ which questions their suitability as potential decompression rates. Supporting this argument are other external findings of note where two pigs presenting with rectal prolapses were those exposed to 40 ms^−1^ and 100 ms^−1^ cycles.

Lesions were observed on both body sides regardless of treatment and this is likely the result of convulsive activity observed during the latter stages of the stun as a result of hypoxic effects on brain function. As with gas stunning using immersion in 80% CO_2_, the anesthetic principle for decompression stunning results from the lack of O_2_ which causes hypoxia (60). Suppressed brain states may lead to behavioral patterns and spontaneous physical reflexes often used as proxies when determining unconsciousness in animals following stun at abattoirs (7). These include tonic rigidity or collapse, apnea, subsequent tonic – clonic seizures and the absence of eye reflexes. The clonic phase can involve paddling and kicking motions which could be responsible for bruising and lesions observed post-mortem. Even though the pigs in this experiment were supported in slings, such seizure-like behaviors were observed (33) where pigs came into contact with the sides of the box in which they were suspended in the chamber. Injuries to the skin on the body and legs are not uncommon in slaughter for the majority of species and are often associated with poor lairage and transport practices as well as understocking or overstocking in CO_2_ stunning gondolas, so that during convulsive activity the animals receive minor bruising and abrasions against equipment or from each other (61–63). Damage as a result of convulsions is not considered a welfare issue as pigs are unconscious during these convulsions, but it does raise concerns related to carcass and meat quality.

### Internal pathology

The detailed pathological examination demonstrated that when pigs are exposed to decompression (and recompression) widespread congestion is apparent in multiple organs. As the pig's body contains a number of gas-filled cavities (thoracic, abdominal), alterations to ambient pressure can restrict escape of gas in these cavities leading to distension. There was no macroscopic evidence of gas bubbles or accumulations in subcutaneous tissues, fascial planes, muscles, organs, blood or joint fluids. There was no evidence of pulmonary emphysema or gas trapped in sub-serosal or sub-mucosal sites, which can cause mechanical damage to tissues and organs (31). The congestion and hemorrhage seen in organs located in these cavities is likely reflecting the body's attempts to equalize pressure. The internal findings of accumulation of ascitic and pericardial serosanguineous fluid was consistent with fluid effusions into these cavities. It is likely this was also a result of the pressure imbalance, with pressure in the cardio-vascular system causing fluid to accumulate and potential heart failure associated with the gradual loss of cardiac function (33). From experience of post-mortem examination of pigs of this age, volumes of fluid normally seen in the pericardium and abdominal cavities would be ~2–4 and 5–8 ml respectively (Thomson pers comm), so the larger volumes seen post-mortem were considered unusual. If large volumes of ascitic fluid is evident when abattoir staff first open the abdomen the carcass is typically diverted onto the detained line for assessment by a meat inspector. However, it should be noted that unlike slaughter line pigs, the pigs in this experiment were not hung and exsanguinated. The gravity during exsanguination post-stun would influence how much fluid would accumulate in cavities; therefore it is unclear whether this is a result of the decompression stunning procedure (including the progressive heart failure) or something that would present regardless of stun type without suspension and exsanguination. There are, to our knowledge, no data available on fluid accumulation in non-exsanguinated pigs from other stunning methods and in general for many of the pathological findings, comparisons with other stunning methods are problematic as there is a lack of equivalent data available. Despite the fluid accumulation, it is important to note that there was no hemorrhage in the heart or abdomen, this could suggest that the cardio-vascular system is functioning as intended (i.e., the heart is maintaining its integrity to ensure blood flow persists to organs). The vascular system is reacting, and the peritoneum is responding to changes in pressure, causing effusion (but no hemorrhage) as it tries to equilibrate. However, fluid effusion is complex and can occur due to cardiac insufficiency due to pathology (e.g., mulberry heart disease) or functional deficits (e.g., ventricular septal defects or heart valve lesions). Whilst the pigs in the study are unlikely to have these conditions (being young animals from a high health status farm, with no clinical signs of health problems), it is important to consider the fluid effusion might not have been solely related to pressure changes, although no cardiac lesions were observed in this study. Hypoxia can also influence the integrity of the vascular system; many tissues as they become starved of oxygen can present with hypoxia/agonal hemorrhage. The endothelium of blood vessels is affected and may leak blood which produces the petechial and ecchymotic hemorrhages which are commonly seen in recently killed animals (64).

Congestion and hemorrhage were observed in both lungs, with higher severity in the right lung in pigs and higher scores observed compared to pathological findings in poultry exposed to decompression (17). The changes seen in the lungs relate to the fact the thoracic cavity is a semi-open, gas-filled cavity, linked to the external environment with a bi-directional lung structure. Therefore, it will be affected by changes in pressure and when the pressure drops it will try to equilibrate. Whether or not this is a welfare issue depends on when this is occurring in relation to loss of consciousness. If the damage occurs before loss of consciousness this could cause concern due to feelings of breathlessness/dyspnoea, which are aversive to pigs and can cause pain (65–67). Pulmonary barotrauma occurs when a pressure differential occurs, which can either result from an increase in external pressure and subsequent decrease in the lungs or vice versa (68). If the lung becomes overdistended it can rupture, which can lead to air embolism, mediastinal emphysema (which can cause cardiovascular compromise), or tension pneumothorax (69). A relevant point made by the veterinary pathologist is that unhealthy lungs could be more affected by changes in pressure. This is also noted in humans experiencing hyperbaric oxygen therapy; blood and oxygen tensions become elevated and therefore sudden changes in already traumatized/injured regions may escalate injuries further (69). Given that respiratory diseases are among the most significant infectious health issues within the pig production industry (10, 20, 70–72) it is an important factor to consider when determining the potential of using gradual decompression as a stunning method. Concerns relating to the health of the pigs presenting at slaughter that might affect the response to decompression have also been discussed by Bouwsema and Lines (9) in their recent review intended to determine the feasibility of LAPS in pigs. They highlight the potential for the paranasal sinuses (another semi-open cavity) to be vulnerable if inflamed such is the case with upper respiratory tract diseases such as atrophic rhinitis. Given this concern it is important to highlight the general trend in our study for the nasal cavities and frontal sinuses to have high congestion and hemorrhage scores for pigs exposed to higher decompression rates. The damage to the nasal cavities including nasal conchae, frontal sinuses and cranium was consistent with lesion scores observed in birds which had undergone decompression at faster commercial rates than those conducted here and this damage was absent in birds killed by anesthetic overdose *via* intravenous injection of pentobarbital sodium (15). As already discussed here and in the poultry work, the timing of this damage is not known and evidence suggests that rapid recompression is associated with trauma in mammals rather than gradual decompression (27–30). However, given the unknowns about when damage is occurring and the known vulnerability of pigs to suffer from respiratory problems it is important to be prudent and assume there could be the potential for welfare detriments.

The lung hemorrhage observed in pigs was more severe than that observed in poultry (15), however a bird's respiratory system, obviously suited to the habit of flight and adapted to respond to pressure changes at altitude, may make it less vulnerable to pressure changes. Where there were similarities in lung changes between the pigs and poultry was the fact that both species presented with higher lesion scores in the right lung (15). This was irrespective of rate or cycle length and is difficult to explain. Similar historical findings where observed in decompression injuries observed in cats (54), but with no explanation. In the current study we postulated it may simply be due to the majority of technical staff being right-handed and therefore when lifting and placing the bodies for transfer for post-mortem they were more likely to position the pigs ventrally on their right side, resulting in pooling of blood and fluids during refrigeration pre-pathological assessment. However, in the poultry studies birds were bled and underwent post-mortem in much shorter periods post decompression and recompression, and such pooling effects were unlikely. It remains an unexplained finding.

The other semi-open cavity of concern is the middle ear. The eustachian tube connects the middle ear and the pharynx. If this is blocked, the air pressure in the middle ear is different to the pressure in the external ear canal and this can cause barotrauma; a condition often reported by humans experiencing high altitudes and a side-effect seen in patients experiencing hyperbaric oxygen therapy (73). This condition is also exacerbated when experiencing nasal congestion or an upper respiratory infection. There was no obvious external damage reported in this current study to ears, but we did not examine the tympanic membrane and as the animals were sedated no behavioral indicators of ear pain could be noted. This will be a focus for further study.

Although congestion scores were recorded there was relatively little damage reported in the gastrointestinal tract and these results are likely reflective of the fact the *gastrointestinal tract* is better able to adjust to pressure changes, essentially being a cavity that is open at both ends. However, it should be noted that gas expansion in the *gastrointestinal tract* could be of concern for conscious animals. In similar work conducted in poultry there was no evidence of intestinal rupture or damage following LAPS, but as these authors noted there could be expansion with welfare implications without pathological consequences (15). Pre-slaughter food withdrawal for ~24 h is common in pigs and was carried out in the current trial for at least 12 h pre-procedure. It is primarily done commercially to improve meat quality and reduce the risk of microbial contamination of carcasses if the GIT is accidently cut during carcass preparation on the production line (74). Food withdrawal also reduces the incidence of pigs presenting with intestinal torsion following transport pre-slaughter, a painful and often fatal condition thought to be exacerbated by stress (75). Excessive gas produced in the intestines (bloat) as a result of irregular feeding, excessive intakes or highly fermentable substrates can lead to intestinal torsion (76). Pigs experiencing gastro-intestinal discomfort can show clinical signs including behaviors indicative of pain (e.g., arched backs, high pitched vocalizations, dog sitting) (77, 78). Thus, behavioral observations in conscious pigs experiencing the stun procedure would be necessary to more fully inform the potential for welfare concerns relating to gut distension.

## Conclusions

The findings of this experiment show that exposure to low atmospheric pressure at the tested rates results in non-recovery in anesthetized pigs. However, there was congestion in most organs and some hemorrhage, which could raise concerns for the welfare of conscious pigs undergoing this type of stunning, depending on when in the cycle the damage is occurring. Although there was limited variation between treatment groups, there was some evidence that the slowest and fastest rates may not be appropriate for application. Whether the pathological damage observed is a response to hypoxia, decompression or recompression cannot be determined from this study, but it is likely that some of the outcomes reflect the cardiovascular response expected when exposed to changes in pressure. At what point this is happening (pre or post loss of consciousness) determines if it is a welfare issue, which cannot be identified from this study. Exsanguination may also affect pathological findings and the long hold times employed may have contributed to the changes observed. We do not have comparative pathological data for other stunning methods including normobaric hypercapnic hypoxia (i.e., CO_2_ stunning). The major limiting factor of this study and any work investigating decompression stunning in animals is the inability to separate decompression from recompression, as recompression is always required to remove the animal from the chamber However it should be noted that cessation of cardiac activity several minutes before recompression should have eliminated any haemodynamic effects relating to cardiac function and blood pressure. In summary, this study provides data on candidate decompression rate parameters to inform future explorations of decompression as a potential stunning method for pigs.

## Data availability statement

The raw data supporting the conclusions of this article will be made available by the authors, without undue reservation.

## Ethics statement

The animal study was reviewed and approved by University of Edinburgh and SRUC Animal Welfare and Ethical Review Bodies (AWERBs, study approval refs: L325 and ED AE14-2018).

## Author contributions

EB, JM, and DM contributed to the conception, funding acquisition, experimental design of the study, and jointly wrote the first draft of the manuscript. JM, MF, JT, and EB all contributed to various levels of the data collection, experimental planning, and practical work. RC, RG, and SG provided veterinary care, anesthesia induction, and maintenance for the pigs. JT conducted all post-mortem pathological assessments. JM conducted all data processing and analysis. All authors contributed to the manuscript revision and final editing. All authors contributed to the article and approved the submitted version.

## Funding

This research was jointly funded by the Department for Environment, Food & Rural Affairs (DEFRA), UK Government (Grant reference. MH0154) and the Humane Slaughter Association (HSA), UK. The Roslin Institute was funded by a BBSRC Institute Strategic Program Grant BB/P013759/1. SRUC receives funding from the Scottish Government's Rural and Environment Science and Analytical Services Division (RESAS). For the purpose of open access, the author has applied a Creative Commons Attribution (CC BY) license to any Author Accepted Manuscript version arising from this submission.

## Conflict of interest

The authors declare that the research was conducted in the absence of any commercial or financial relationships that could be construed as a potential conflict of interest.

## Publisher's note

All claims expressed in this article are solely those of the authors and do not necessarily represent those of their affiliated organizations, or those of the publisher, the editors and the reviewers. Any product that may be evaluated in this article, or claim that may be made by its manufacturer, is not guaranteed or endorsed by the publisher.
