# Supplementary material for: Characterizing candidate decompression rates for hypobaric hypoxic stunning of pigs. Part 2: Pathological consequences
Source: Front Vet Sci. 2022 Nov 9;9:1027883. doi: 10.3389/fvets.2022.1027883 (PMC9681787; doi:10.3389/fvets.2022.1027883)
Supplement: Supplementary file 1 [file Data_Sheet_1.docx]

Supplementary Material

# Supplementary Figures and Tables

**Table S1:** Counts of pigs observed across pathological scores (0-5) for congestion and hemorrhage within the head and neck regions of the animal. A total of six pigs underwent detailed pathological assessment within each target decompression treatment [pathological scoring criteria: 0 = no change; 1 = a very slight but noticeable change; 2 = a low grade change; 3 = a moderate change; 4 = a marked change; and 5 = a severe change].

| **Anatomical area** | **Cycle rate (ms^-1^) and length (s)** | **Congestion score** | | | | | | **Haemorrhage score** | | | | | | |
| --- | --- | --- | --- | --- | --- | --- | --- | --- | --- | --- | --- | --- | --- | --- |
|  |  | **0** | **1** | **2** | **3** | **4** | **5** | **0** | **1** | **2** | **3** | **4** | **5** |  |
| Ears | 40 (720s) | 2 | 3 | 1 |  |  |  | 6 |  |  |  |  |  |  |
|  | 60 (720s) |  |  | 3 | 3 |  |  | 6 |  |  |  |  |  |  |
|  | 60 (480s) |  | 1 | 5 |  |  |  | 6 |  |  |  |  |  |  |
|  | 80 (720s) |  | 2 | 2 | 2 |  |  | 6 |  |  |  |  |  |  |
|  | 100 (720s) | 1 | 3 | 1 | 1 |  |  | 6 |  |  |  |  |  |  |
| Oral cavity & tongue | 40 (720s) |  | 5 |  | 1 |  |  | 6 |  |  |  |  |  |  |
|  | 60 (720s) | 2 | 1 | 3 |  |  |  | 6 |  |  |  |  |  |  |
|  | 60 (480s) | 2 | 1 | 1 | 2 |  |  | 6 |  |  |  |  |  |  |
|  | 80 (720s) | 4 | 1 | 1 |  |  |  | 6 |  |  |  |  |  |  |
|  | 100 (720s) | 1 | 4 |  | 1 |  |  | 6 |  |  |  |  |  |  |
| Teeth (periodontal tissues) | 40 (720s) | 6 |  |  |  |  |  | 6 |  |  |  |  |  |  |
|  | 60 (720s) | 6 |  |  |  |  |  | 6 |  |  |  |  |  |  |
|  | 60 (480s) | 6 |  |  |  |  |  | 6 |  |  |  |  |  |  |
|  | 80 (720s) | 6 |  |  |  |  |  | 6 |  |  |  |  |  |  |
|  | 100 (720s) | 6 |  |  |  |  |  | 6 |  |  |  |  |  |  |
| Conjunctivae | 40 (720s) |  |  | 2 | 2 | 2 |  | 3 | 2 |  | 1 |  |  |  |
|  | 60 (720s) |  | 1 | 1 |  | 4 |  | 3 | 3 |  |  |  |  |  |
|  | 60 (480s) |  |  | 2 | 2 | 2 |  | 4 | 2 |  |  |  |  |  |
|  | 80 (720s) |  | 2 | 1 | 2 | 1 |  | 4 | 2 |  |  |  |  |  |
|  | 100 (720s) |  | 3 | 2 | 1 |  |  | 3 | 2 |  |  |  | 1 |  |
| Sclera | 40 (720s) | 2 |  | 1 | 2 | 2 |  | 6 |  |  |  |  |  |  |
|  | 60 (720s) |  | 1 | 1 |  | 4 |  | 6 |  |  |  |  |  |  |
|  | 60 (480s) | 3 |  |  | 1 | 2 |  | 6 |  |  |  |  |  |  |
|  | 80 (720s) |  |  | 1 | 3 | 2 |  | 6 |  |  |  |  |  |  |
|  | 100 (720s) | 2 |  | 1 | 1 | 1 | 1 | 5 | 1 |  |  |  |  |  |
| Nasal planum | 40 (720s) | 3 | 3 |  |  |  |  | 6 |  |  |  |  |  |  |
|  | 60 (720s) | 1 | 1 | 3 | 1 |  |  | 6 |  |  |  |  |  |  |
|  | 60 (480s) | 1 | 1 | 2 | 1 | 1 |  | 6 |  |  |  |  |  |  |
|  | 80 (720s) | 2 | 1 | 3 |  |  |  | 6 |  |  |  |  |  |  |
|  | 100 (720s) | 3 | 1 | 2 |  |  |  | 6 |  |  |  |  |  |  |
| Frontal sinuses | 40 (720s) | 3 | 2 | 1 |  |  |  | 2 | 4 |  |  |  |  |  |
|  | 60 (720s) | 2 | 1 | 2 |  | 1 |  | 1 | 1 | 3 | 1 |  |  |  |
|  | 60 (480s) |  | 1 | 3 | 2 |  |  | 2 | 2 | 2 |  |  |  |  |
|  | 80 (720s) | 3 | 1 | 2 |  |  |  | 1 | 3 | 2 |  |  |  |  |
|  | 100 (720s) |  | 2 | 2 |  | 2 |  | 2 | 2 | 2 |  |  |  |  |
| Nasal cavities and conchae | 40 (720s) |  |  | 2 |  |  |  | 5 | 1 |  |  |  |  |  |
|  | 60 (720s) |  |  | 3 | 1 | 2 |  | 5 | 1 |  |  |  |  |  |
|  | 60 (480s) |  |  |  | 5 | 1 |  | 1 |  | 5 |  |  |  |  |
|  | 80 (720s) | 1 |  | 1 | 3 | 1 |  | 5 |  | 1 |  |  |  |  |
|  | 100 (720s) |  | 1 | 1 | 2 | 2 |  | 6 |  |  |  |  |  |  |
| Cranium | 40 (720s) | 3 | 2 | 1 |  |  |  | 5 | 1 |  |  |  |  |  |
|  | 60 (720s) | 2 | 2 | 2 |  |  |  | 5 | 1 |  |  |  |  |  |
|  | 60 (480s) |  | 4 | 1 | 1 |  |  | 5 | 1 |  |  |  |  |  |
|  | 80 (720s) | 3 | 1 | 1 | 1 |  |  | 6 |  |  |  |  |  |  |
|  | 100 (720s) | 2 | 3 | 1 |  |  |  | 5 |  | 1 |  |  |  |  |
| Meninges and brain | 40 (720s) | 1 | 1 | 3 | 1 |  |  | 5 | 1 |  |  |  |  |  |
|  | 60 (720s) |  |  | 5 |  | 1 |  | 5 |  | 1 |  |  |  |  |
|  | 60 (480s) |  |  | 3 | 3 |  |  | 5 | 1 |  |  |  |  |  |
|  | 80 (720s) |  | 1 | 4 | 1 |  |  | 5 | 1 |  |  |  |  |  |
|  | 100 (720s) |  |  | 6 |  |  |  | 6 |  |  |  |  |  |  |
| Trachea | 40 (720s) | 5 | 1 |  |  |  |  | 5 | 1 |  |  |  |  |  |
|  | 60 (720s) | 5 | 1 |  |  |  |  | 5 |  | 1 |  |  |  |  |
|  | 60 (480s) | 4 | 1 | 1 |  |  |  | 4 | 1 | 1 |  |  |  |  |
|  | 80 (720s) | 6 |  |  |  |  |  | 5 | 1 |  |  |  |  |  |
|  | 100 (720s) | 4 | 2 |  |  |  |  | 6 |  |  |  |  |  |  |
| Cervical tissues | 40 (720s) | 6 |  |  |  |  |  | 6 |  |  |  |  |  |  |
|  | 60 (720s) | 6 |  |  |  |  |  | 5 | 1 |  |  |  |  |  |
|  | 60 (480s) | 6 |  |  |  |  |  | 6 |  |  |  |  |  |  |
|  | 80 (720s) | 6 |  |  |  |  |  | 6 |  |  |  |  |  |  |
|  | 100 (720s) | 6 |  |  |  |  |  | 6 |  |  |  |  |  |  |

**Table S2:** Counts of pigs observed across pathological scores (0-5) for congestion and hemorrhage within the trunk and extremities of the animal. A total of six pigs underwent detailed pathological assessment within each target decompression treatment [pathological scoring criteria: 0 = no change; 1 = a very slight but noticeable change; 2 = a low grade change; 3 = a moderate change; 4 = a marked change; and 5 = a severe change].

| **Anatomical area** | **Cycle rate (ms^-1^) and length (s)** | **Congestion score** | | | | | | **Haemorrhage score** | | | | | |
| --- | --- | --- | --- | --- | --- | --- | --- | --- | --- | --- | --- | --- | --- |
|  |  | **0** | **1** | **2** | **3** | **4** | **5** | **0** | **1** | **2** | **3** | **4** | **5** |
| Legs (skin, muscles and connective tissues) | 40 (720s) | 6 |  |  |  |  |  | 6 |  |  |  |  |  |
|  | 60 (720s) | 6 |  |  |  |  |  | 5 |  | 1 |  |  |  |
|  | 60 (480s) | 6 |  |  |  |  |  | 5 |  |  | 1 |  |  |
|  | 80 (720s) | 6 |  |  |  |  |  | 5 | 1 |  |  |  |  |
|  | 100 (720s) | 6 |  |  |  |  |  | 5 | 1 |  |  |  |  |
| External orifices ( anus, vulva) | 40 (720s) | 5 |  |  |  | 1 |  | 5 | 1 |  |  |  |  |
|  | 60 (720s) | 6 |  |  |  |  |  | 6 |  |  |  |  |  |
|  | 60 (480s) | 6 |  |  |  |  |  | 6 |  |  |  |  |  |
|  | 80 (720s) | 6 |  |  |  |  |  | 3 | 3 |  |  |  |  |
|  | 100 (720s) | 5 | 1 |  |  |  |  | 3 | 2 |  | 1 |  |  |
| Representative joint cavities (stifles and elbows) | 40 (720s) | 5 |  |  | 1 |  |  | 6 |  |  |  |  |  |
|  | 60 (720s) | 5 |  | 1 |  |  |  | 6 |  |  |  |  |  |
|  | 60 (480s) | 6 |  |  |  |  |  | 6 |  |  |  |  |  |
|  | 80 (720s) | 6 |  |  |  |  |  | 5 | 1 |  |  |  |  |
|  | 100 (720s) | 6 |  |  |  |  |  | 6 |  |  |  |  |  |
| Liver | 40 (720s) |  |  | 1 | 4 | 1 |  | 5 | 1 |  |  |  |  |
|  | 60 (720s) |  | 1 | 3 | 2 |  |  | 6 |  |  |  |  |  |
|  | 60 (480s) |  |  |  | 6 |  |  | 6 |  |  |  |  |  |
|  | 80 (720s) |  |  | 1 | 5 |  |  | 6 |  |  |  |  |  |
|  | 100 (720s) |  | 1 |  | 5 |  |  | 6 |  |  |  |  |  |
| Spleen | 40 (720s) |  |  | 5 |  | 1 |  | 5 |  | 1 |  |  |  |
|  | 60 (720s) |  | 1 | 2 | 3 |  |  | 6 |  |  |  |  |  |
|  | 60 (480s) |  |  | 3 | 3 |  |  | 6 |  |  |  |  |  |
|  | 80 (720s) |  |  | 3 | 3 |  |  | 5 |  | 1 |  |  |  |
|  | 100 (720s) |  |  | 5 | 1 |  |  | 6 |  |  |  |  |  |
| Heart | 40 (720s) | 4 | 2 |  |  |  |  | 6 |  |  |  |  |  |
|  | 60 (720s) | 5 | 1 |  |  |  |  | 6 |  |  |  |  |  |
|  | 60 (480s) | 5 | 1 |  |  |  |  | 6 |  |  |  |  |  |
|  | 80 (720s) | 6 |  |  |  |  |  | 6 |  |  |  |  |  |
|  | 100 (720s) | 3 | 2 | 1 |  |  |  | 6 |  |  |  |  |  |
| Left lung | 40 (720s) |  |  | 3 | 1 | 1 | 1 | 3 | 1 | 1 |  | 1 |  |
|  | 60 (720s) |  |  | 2 | 3 |  | 1 | 2 | 1 | 3 |  |  |  |
|  | 60 (480s) |  |  | 2 | 1 | 3 |  |  | 3 | 1 |  |  | 2 |
|  | 80 (720s) |  |  |  | 3 | 3 |  | 2 | 2 | 1 | 1 |  |  |
|  | 100 (720s) |  |  |  | 3 | 3 |  | 2 | 1 | 3 |  |  |  |
| Right lung | 40 (720s) |  |  | 3 | 1 | 1 | 1 | 3 | 1 | 1 |  | 1 |  |
|  | 60 (720s) |  |  | 3 | 1 |  | 2 | 2 | 2 | 1 |  | 1 |  |
|  | 60 (480s) |  |  | 1 | 1 | 4 |  |  | 2 | 2 |  |  | 2 |
|  | 80 (720s) |  |  |  | 4 | 2 |  | 2 | 2 | 1 | 1 |  |  |
|  | 100 (720s) |  | 1 |  | 3 | 2 |  | 3 | 1 | 2 |  |  |  |
| Cranial thoracic cavity | 40 (720s) | 6 |  |  |  |  |  | 5 |  |  | 1 |  |  |
|  | 60 (720s) | 6 |  |  |  |  |  | 6 |  |  |  |  |  |
|  | 60 (480s) | 6 |  |  |  |  |  | 6 |  |  |  |  |  |
|  | 80 (720s) | 6 |  |  |  |  |  | 6 |  |  |  |  |  |
|  | 100 (720s) | 6 |  |  |  |  |  | 6 |  |  |  |  |  |
| Caudal thoracic cavity | 40 (720s) | 5 |  |  | 1 |  |  | 5 |  |  | 1 |  |  |
|  | 60 (720s) | 6 |  |  |  |  |  | 6 |  |  |  |  |  |
|  | 60 (480s) | 6 |  |  |  |  |  | 6 |  |  |  |  |  |
|  | 80 (720s) | 6 |  |  |  |  |  | 6 |  |  |  |  |  |
|  | 100 (720s) | 6 |  |  |  |  |  | 6 |  |  |  |  |  |
| Abdominal cavity | 40 (720s) | 5 |  |  | 1 |  |  | 6 |  |  |  |  |  |
|  | 60 (720s) | 6 |  |  |  |  |  | 6 |  |  |  |  |  |
|  | 60 (480s) | 5 | 1 |  |  |  |  | 6 |  |  |  |  |  |
|  | 80 (720s) | 5 |  |  | 1 |  |  | 6 |  |  |  |  |  |
|  | 100 (720s) | 6 |  |  |  |  |  | 6 |  |  |  |  |  |
| Duodenum & small intestine | 40 (720s) |  |  | 3 | 3 |  |  | 6 |  |  |  |  |  |
|  | 60 (720s) |  |  | 1 | 5 |  |  | 6 |  |  |  |  |  |
|  | 60 (480s) |  |  |  | 2 | 4 |  | 6 |  |  |  |  |  |
|  | 80 (720s) |  |  |  | 5 | 1 |  | 6 |  |  |  |  |  |
|  | 100 (720s) |  |  | 1 | 4 | 1 |  | 6 |  |  |  |  |  |
| Pancreas | 40 (720s) | 6 |  |  |  |  |  | 6 |  |  |  |  |  |
|  | 60 (720s) | 6 |  |  |  |  |  | 6 |  |  |  |  |  |
|  | 60 (480s) | 6 |  |  |  |  |  | 6 |  |  |  |  |  |
|  | 80 (720s) | 6 |  |  |  |  |  | 6 |  |  |  |  |  |
|  | 100 (720s) | 6 |  |  |  |  |  | 6 |  |  |  |  |  |
| Caecum and colon | 40 (720s) |  | 2 | 2 | 2 |  |  | 6 |  |  |  |  |  |
|  | 60 (720s) |  | 2 | 2 | 2 |  |  | 6 |  |  |  |  |  |
|  | 60 (480s) |  |  | 5 | 1 |  |  | 6 |  |  |  |  |  |
|  | 80 (720s) |  | 1 | 2 | 3 |  |  | 6 |  |  |  |  |  |
|  | 100 (720s) |  |  | 1 | 5 |  |  | 6 |  |  |  |  |  |
| Kidney | 40 (720s) |  |  | 1 | 4 | 1 |  | 5 |  | 1 |  |  |  |
|  | 60 (720s) |  |  | 2 | 4 |  |  | 5 |  |  | 1 |  |  |
|  | 60 (480s) |  |  | 1 | 5 |  |  | 4 |  | 1 | 1 |  |  |
|  | 80 (720s) |  |  | 1 | 4 | 1 |  | 6 |  |  |  |  |  |
|  | 100 (720s) |  |  | 1 | 5 |  |  | 5 |  |  | 1 |  |  |
| Stomach | 40 (720s) | 6 |  |  |  |  |  | 6 |  |  |  |  |  |
|  | 60 (720s) | 6 |  |  |  |  |  | 6 |  |  |  |  |  |
|  | 60 (480s) | 5 | 1 |  |  |  |  | 6 |  |  |  |  |  |
|  | 80 (720s) | 6 |  |  |  |  |  | 6 |  |  |  |  |  |
|  | 100 (720s) | 5 |  | 1 |  |  |  | 6 |  |  |  |  |  |
